# Potential role of salivary vitamin D antimicrobial peptide LL-37 and interleukins in severity of dental caries: an exvivo study

**DOI:** 10.1186/s12903-023-03749-7

**Published:** 2024-01-13

**Authors:** Nireeksha, Mithra N Hegde, Suchetha Kumari N

**Affiliations:** 1 Department of Conservative Dentistry and Endodontics, AB Shetty Memorial Institute of Dental Sciences, Nitte (deemed to be) University, Mangaluru, India; 2 Department of Biochemistry, KS Hegde Medical Academy, Nitte (deemed to be) University, Mangaluru, India

**Keywords:** Vitamin D, Dental caries, Antimicrobial peptides, Interleukins, Ex-vivo

## Abstract

**Introduction:**

Vitamin D performs various functions as a hormone by promoting calcium absorption but plays a major role in innate immunity,cell differentiation, cell maturation through its genomic effects via vitamin D receptor. The immune response also plays a major role in tooth surface and supporting structure destruction and playing a major factor in high caries formation. The inflammatory cytokines are released has proinflammatory cytokines and stimulate cells in disease process. Therefore, in the present study we have evaluated the association of salivary vitamin D, LL-37, interleukins 6 and 17A in various levels of severity of dental caries.

**Method:**

Ethical approval was obtained (NU/CEC/2020/0339), 377 individuals reporting to department of conservative dentistry and endodontics, AB Shetty memorial institute of dental sciences were included based on inclusion criteria. The individuals were further divided into caries free(*N* = 105) and caries active(*N* = 272) based on their caries prevalence. The salivary were collected and evaluated for vitamin D, LL-37,IL-17A and IL-6.Results were statistically analysed with SPSS vs 22 (IBM Corp, USA). Normally distributed data were expressed as mean ± SD. Skewed data were expressed as median and interquartile range. To compare (mean) outcome measures between the two groups unpaired independent t-test was applied and for values in median IQR, Mann Whitney U test was used. All statistical analysis for *P* value were two-sided and significance was set to *P* ≤ 0.05.

**Results:**

The study showed that, the salivary vitamin D statistically decreased with increasing severity of caries which showed that vitamin D plays an important role in prevention of caries. Antimicrobial peptide LL-37 was higher in caries free group but was not statistically significant, salivary IL-6 level was higher in caries active group but intergroup comparison did not show significant difference. Salivary IL-17A did not show statistically significant between caries active and caries free group.

**Conclusion:**

The salivary levels of vitamin D may play a vital role in prevalence of dental caries and its severity which can be a underlying cause in presence of other etiological factors.

## Introduction

Vitamin D deficiency is one of the most ignored and undiagnosed conditions in the general population [1, 2]. Studies conducted in the past among various communities and locations of Indian population exhibited prevalence of Vitamin D deficiencies ranging from 50 to 94%, which was indicative of the magnitude of the problem in the country. These deficiencies are associated with individuals with detected systemic illness therefore [3].The major causes for vitamin D deficiency in Indian population can be attributed to a low vitamin D dietary intake, increased indoor lifestyle, decreased exposure to sunlight, and increased air pollution which in turn hampers synthesis of Vitamin D by the skin after absorption of UV rays [4, 5]. Past research has shown that it is imperative to address Vitamin D deficiency and correlate it to systemic health issues since Vitamin D deficiency causes autoimmune diseases, infectious diseases, cancer, skeletal manifestation, and depression [6]. Research has also shown that Vitamin D deficiency leads to problems in oral health viz. poor tooth formation, development and calcification in younger adults, poor periodontal health, and malignant oral lesions [7].

**Fig. 1:**
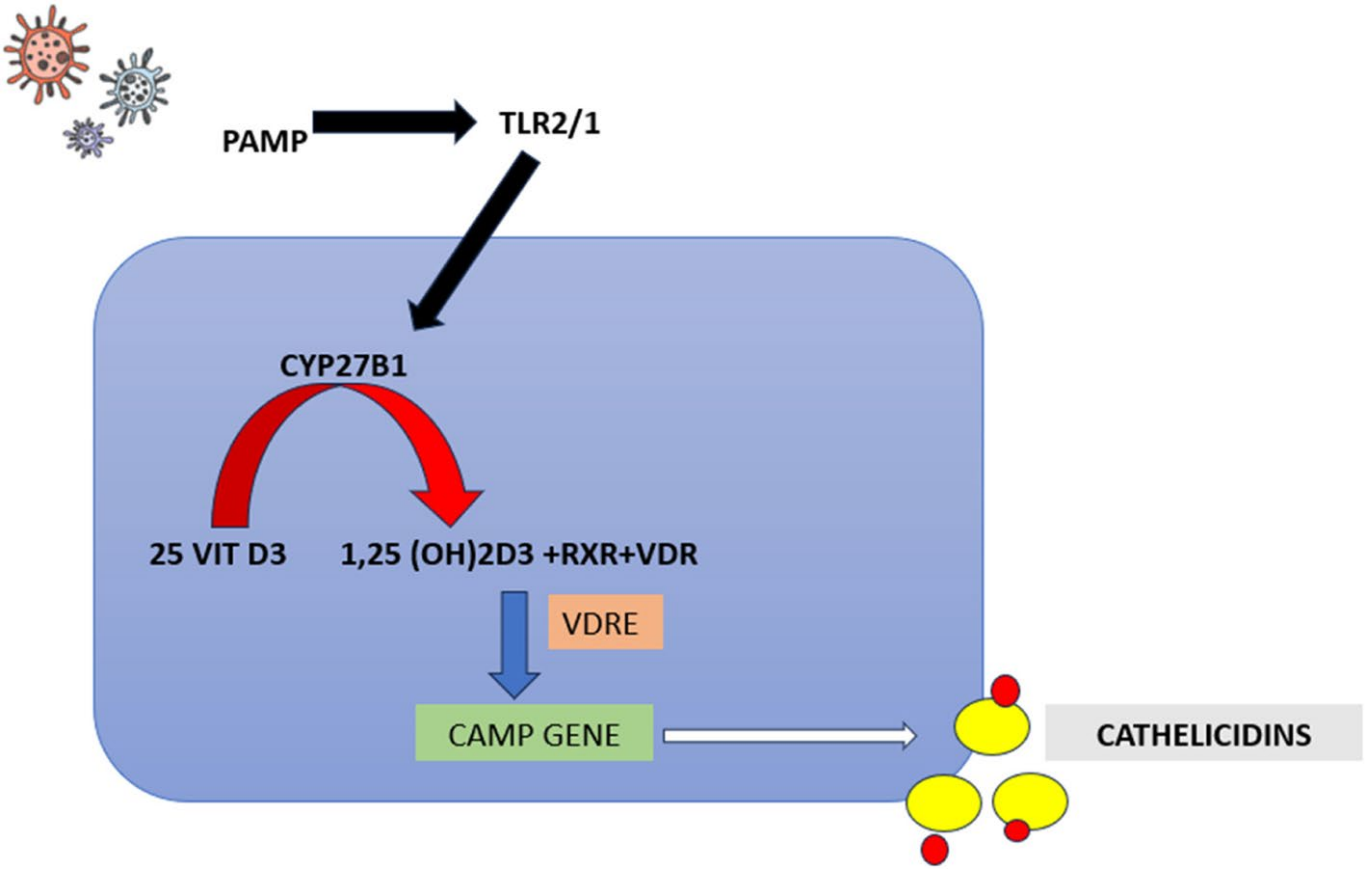
Immunomodulatory role of vitamin D through production of antimicrobial peptides. Presence pathogenic microorganisms stimulate the activation of TLR1/2 and stimulate CYP27B1, which in turn converts the inactive vitamin D to active vitamin D; leading to complexation with RXR and VDR. This whole complex adheres to the Vitamin D response element on the CAMP gene to produce Cathelicidins/LL-37

Vitamin D is also plays a vital role in innate immune responses by promoting immune cell differentiation and cell maturation. Active Vitamin D is absorbed onto the Vitamin D receptors (VDR) on the immune cells, which in turn promote gene expression and regulation of protective peptides [8]. This non-classical action of the VDR and CYP27B1 expressed on various cells and tissues are not associated with calcium haemostasis but are instead dependent on pathogen detection and cytokine production via interleukin production. The circulating vitamin D 1,25-dihydroxy vitamin forms complexes with the Retinoid X receptor vitamin D binding protein complex (RXR + VDBP) to attach to the VDRs on various target cells and tissues. Finally, this complex attaches to the vitamin D promoter regions on the vitamin D receptor genes in order to stimulate the production of the antimicrobial peptide LL-37 [9, 10]. Vitamin D regulates the innate immune response via production of these peptides in various concentrations in body fluids [11] (illustrated in Fig. 1).

The cytokines released during this immune response contains pro-inflammatory cytokines to stimulate the cells, which in turn undergo an immuno-modulatory response. Vitamin D also affects to regulation of T-helper cells by contributing to anti-inflammatory effects. Interleukin IL-17A is produced by Th17A, which in turn regulates NF-KB and other nitrogen activated protein kinases to regulate the IL-6 expression. IL-6 is an important interleukin to promote host defence and immune response, especially in the oral cavity where an absence of the immune response leads to destruction of tooth structure and causing caries [12]. Keeping this in mind, the present study evaluated the association of salivary Vitamin D levels and the levels of LL-37, IL-6, and IL-17A in the severity of dental caries.

## Materials and methods

For conducting the present study, necessary approvals were obtained from the Central ethics committee, Nitte (deemed to be) University. Approval obtained dated NU/CEC/2020/0339 and NU/CEC/2022/291, prior initiation of the study, also renewal done.

Informed consent was obtained from each individual patient, after they were provided with the patient information sheet.The experimental protocols were approved by the scientific committee prior to the commencement of the study. A total of 377 patients who visited the outpatient department of conservative dentistry and endodontics at the A.B. Shetty Memorial Institute of Dental Services, Deralakatte, Mangalore, were included in the study. Out of these, 272 patients were designated as Caries active and 105 were designated as Caries free.

The Sample size (N) was calculated estimating the difference between two means and by using the formula presented below.

$${\text{N}}=\frac{{\left({{\text{Z}}}_{1-\mathrm{\alpha }/2}+{{\text{Z}}}_{\upbeta }\right)}^{2}\left[{{\text{P}}}_{1}\left(1-{{\text{P}}}_{1}\right)+{{\text{P}}}_{2}\left(1-{{\text{P}}}_{2}\right)\right]}{{{\text{P}}}_{1}-{{\text{P}}}_{2}}$$

Where, P1 was the proportion of the 1st group (39%), P2 was the proportion of the 2nd group (24%), α was the significance level (5%), and β was the power of the test (20%).

All the laboratory work was conducted at the Central Research Laboratory, K.S. Hegde Medical Academy, Deralakatte, Mangalore. The time period of the study was from August 2018 to January 2022 (4 years). During the study, all patients were evaluated and informed consent was obtained from them for the purpose of the study using an information sheet. Designation as Caries Free and Caries Active was done based on certain inclusion and exclusion criteria. The inclusion criteria pertained to individuals in the age group of 18–40 years who were not exhibiting symptoms of any systemic and/or local illnesses that could potentially hamper salivary flow. Those individuals who were following a restricted diet, exhibiting symptoms of generalized gingivitis or periodontitis, undergoing long-term medication, having poor oral hygiene habits, who were chronic smokers and/or alcoholics, and individuals consuming specific nutritional supplements were also excluded from the study.

For evaluating the presence of caries, patients were seated in a dental chair and under ideal illumination and evaluated using a mouth mirror and straight probe. In order to create a DMFT index (Decayed, Missing, and Filled Teeth), the WHO Oral health survey format Annexure 1 was used [13]. Individuals were then divided into two groups based on prevalence of caries as well as their DMFT score. The Caries Free group had a DMFT score of 0 while the Caries Active group had DMFT scores ranging from 1–10. The individuals in the Caries Active Group were further subdivided into Decay group 1 (1–3 caries), Decay Group 2 (4–10 caries), and Decay group 3 (> 10 caries).

The general information of the individuals like age, sex, dietary habits like frequency of food intake, type of food habits like non-vegetarian/vegetarian diet, and their brushing habits were also obtained and noted in addition to their medical history.

Following the generation of the DMFT index, a PUFA Index (Pulpal involvement, Ulceration, Fistula, and Abcess) [14] was recorded to delineate the oral conditions in the individuals who were Caries Active. Based on visible root pulp, ulceration of oral mucosa from root fragments, appearance of a fistula or abscess, a score was assigned and recorded.

Pulpal involvement (P/p) was recorded when individuals were noted to have a visible opening of the pulp chamber in their teeth due to caries, thereby leaving only the roots and root fragments. Ulceration (U/u) was recorded in individuals who exhibited significant sharp object trauma from either a broken/dislocated tooth or root fragments as a result of caries. Fistula (F/f) was recorded in those individuals where the pulpal involvement was accompanied by pus releasing sinus tract. Finally, Abscess (A/a) was recorded in those individuals who exhibited a pus contained swelling as a result of pulpal involvement.

In order to collect saliva from the individuals, the Navazesh protocol was used [15]. Individuals were informed to abstain from eating or drinking, brushing their teeth, using mouthwash, or smoking two hours prior to salivary sample collection. Samples were collected between 10 and 11 a.m. In order to maintain a stress free atmosphere and not to hinder salivary flow, the individuals were seated in regular chairs. A Tarson's saliva collection tube was used to collect 5 ml of saliva that had gathered on the floor of the mouth of the individuals. The collected saliva was then centrifuged and the supernatant was stored at -20 °C till further analysis.

### Analysis of salivary vitamin D levels

The analysis of salivary Vitamin D levels was carried out using the 25OH Vitamin D Total ELISA Kit Microtiter Plates (Epitope Diagnostics). 20µL of sample, calibrators, and controls were added to the wells of the plate along with 100µL of the Vitamin D Assay buffer. The plates were covered with aluminium foil and static incubated at room temperature for 30 min. Following this, 25µL of Biotinylated Vitamin D analog was added to each well and static incubated at room temperature for 1 h. Then, each well was washed 5 times with 350µL of the wash solution. This was followed by addition of 100µL Streptavidin Horseradish Peroxidase (HRP) and static incubated at room temperature for 30 min to form the Vitamin D antibody – Vitamin D, Biotin D and HRP conjugated streptavidin complex. The unbound complexes were removed from the wells by washing them five times with 350µL buffer solution and 100µL of tetramethylbenzidine (TMB) was added. The plates were static incubated one final time for 20 min after which 100µL of the stop solution was added. Finally, the reaction mixture was measured spectrophotometrically at 450 nm absorbance with a maximum absorbance time of 10 min.

### Analysis of salivary Cathelicidin levels

For the analysis of the salivary cathelicidins levels, a pre-coated micro-ELISA plate containing the human LL-37-specific antibody (Sincere Biotech) was used. Controls and samples were loaded into the wells of the ELISA plate along with the specific antibody and incubated at room temperature. Following this, the biotinylated detection antibody (specific to human LL-37) and avidin conjugated HRP were added to the wells and incubated once again in static condition. This was followed by a washing step to remove the unconjugated complexes, following which a substrate was added to each well. Those wells in which the complexation occurred turned blue. A final stop solution was added to halt the reaction and the optical density was measured spectrophotometrically at 450 ± 2 nm.

### Analysis of salivary IL-6, IL-17A levels

The analysis of IL-17A and IL-6 were done using commercially available ELISA kits (Booster Biologicals). For the IL-17A, the principle used was the Solid Phase Sandwich ELISA. The samples and standards were added to the wells of the ELISA microtiter plates, facilitating the binding of IL-17A to the immobilized antibodies. Following a washing step, HRP conjugated anti-IL-17A antibody solution was added to the wells, creating an antibody-antigen–antibody sandwich in the process. TMB substrate solution was added to the ‘sandwich’ and incubated followed by stopping the reaction using a stop solution. Finally, the absorbance was measured spectrophotometrically at 620 nm. by booster biological technology.

Similar to IL-17A, a sandwich ELISA approach was also used to measure the IL-6 levels. The microtiter ELISA plates contained immobilized rat monoclonal antibodies, to which standards and samples were added to facilitate the binding of the IL-6. Following, this, a anti-IL-6 antibody was added to create the antibody-antigen–antibody sandwich. After a period of incubation, HRP conjugated streptavidin was added to the wells and incubated. Following a wash step to remove unconjugated elements, TMB was added to the wells followed by a stop solution. The final absorbance was measured spectrophotmetrically at 450 nm and absorbance of samples were compared to the standards.

#### Statistical analysis

Qualitative statistical analysis was performed on the collected data pertaining to frequency, percentage, mean, and standard deviation. A chi square test was performed for comparing the salivary parameters between Caries Active and Caries Free groups. Analysis of Variance (ANOVA) and t-test were also performed for the two groups. The Receiver Operating Characteristic analysis was performed in order to obtain the optimum cut off levels of sensitivity and specificity for Salivary Vitamin D, LL-37, IL-17A and IL-6. SPSS (version 23; IBM SPSS Corp, Armonk, NY, USA) software was used to perform the statistical comparisons and all the statistical analyses for the *P* value were observed to be two-sided. The significance level was set to *P* ≤ 0.05 in order to eliminate overfitting of data.

## Results

### Demographic characteristics

Of the 272 Caries Active and 105 Caries Free individuals, 239 were females and 138 were males (Tables 1 and 2). Data highlighted in Tables 10 and 11 exhibits the comparison between the demographic data and decay groups. Based on the demographics, a significant population of individuals were from urban areas. When decay groups were compared with the demographic data like age groups, sex, location and gender, individuals from urban population showed significant correlation with the decay group 1(i.e., 1–3 caries) with *p* value of 0.011. From the data in Table 2, it can be observed that individuals in the age group 18–25 years were associated to Decay group 1 (1–3 caries). It can also be observed that the 26–35 years age group were closely associated with Decay group 2 (4–10 caries) and Decay group 3 (> 10 caries). For the individuals hailing from urban areas, the PUFA score was observed to be 0 (*p* = 0.0) (data highlighted in Table 3).

**Table 1:** Depicts the association of gender, location, diet and age groups with  caries active group

Characteristics		Caries active group		
		**Group 1**	**Group 2 and 3**	***P* Value**
Gender	Female	34.3%	65.7%	0.346
	Male	40.0%	60.0%	
Diet	Non veg	35.0%	65.0%	0.130
	vegetarian	50.0%	50.0%	
Location	Other	26.3%	73.7%	.011
	Urban	41.8%	58.2%	
Age group	18- 25Y	42.6%	57.4%	.003
	26-35Y	17.0%	83.0%	
	36- 40 Y	28.6%	71.4%

**Table 2:** Depicts the association of demographic data with caries active group and caries free group

Characteristics	Caries active	Caries free	*P* Value
Gender			0.917
Male	36.8%	36.2%	
Female	63.2%	63.8%	
Location			.000
Urban	65.1%	87.6%	
Other (Semiurban, rural)	34.9%	12.4%	
Diet			0.186
Vegetarian	9.6%	14.3%	
Non-vegetarian	90.4%	85.7%	
Age group			0.835
18-25Y	69.9%	66.7%	
26-35Y	17.3%	19.0%	
36-40Y	12.9%	14.3%	

*P* < 0.05 was considered statistically significant

**Table 3:** Depicts association of demographic data with PUFA scores among caries active individuals

Characteristics		PUFA INDEX			*P* value
		**Caries active (pufa score = 1)**		**Caries active (pufa score = 2–5)**	
		**ROW N %**		**ROW N %**	
Gender	Female	7.0%		7.0%	0.006
	Male	19.0%		3.0%	
Diet	Non veg	11.8%		6.1%	0.326
	Vegetarian	7.7%		0.0%	
Location	Other	22.1%		7.4%	.001
	Urban	5.7%		4.5%	
Age group	18- 25y	9.5%		3.2%	.028
	26-35y	19.1%		10.6%	
	36- 40 y	11.4%		11.4%	

### Evaluation of salivary antimicrobial peptide LL-37,Vitamin D, IL6, IL-17A levels in dental caries

Among the individuals classified in the caries active group, the mean salivary vitamin D level was observed to be 20.85 pg/ml in comparison to the significantly higher (*p* < 0.001) 28.56 pg/ml for the individuals in the caries free group (Table 4). It was also observed that the mean salivary Vitamin D decreased with increasing severity of caries in the individuals. In the different subgroups of the Caries Active group, mean salivary vitamin D levels of 16.31 pg/ml was observed in decay group 2 and 3, whereas decay group 1 had a mean salivary vitamin D level of 28.77 pg/ml, which was significantly higher (*p* = 0.00). When the PUFA index scores were correlated to the salivary vitamin D levels, it was observed that the salivary vitamin D was significantly lower (13.46 pg/ml, *p* = 0.026) in individuals with PUFA score of 2–5 when compared to individuals with a PUFA score of 1 (21.13 pg/ml) (data highlighted in Table 5).

**Table 4:** Association of salivary vitamin D with study groups

	N	Mean	Std. Deviation	95% Confidence Interval for Mean		t test *p* value
				Lower Bound	Upper Bound	
Caries free	105	28.56	10.42	26.54	30.58	0.000
Caries active	272	20.85	11.20	19.51	22.18	
Total	377	23.00	11.51	21.83	24.16	

*P* < 0.05 was considered statistically significant

**Table 5:** Represents comparison of salivary levels of vitamin D with decay groups and PUFA scores

	Parameter	Groups	Mean ± S. D	Significance (*P* Value)
Salivary Vitamin D (pg/ml)	Decay Group	1	28.77 ± 10.50	0.000
		2 – 3	16.31 ± 8.83	
	PUFA Score	0	21.13 ± 11.11	0.026
		1	22.44 ± 12.14	
		2 – 5	13.46 ± 8.53	

*P* < 0.05 was considered statistically significant

The logistic regression performed to establish the odds ratio depicted 0.939 effect of salivary vitamin D on caries active group. A 1 unit decrease in salivary vitamin D levels meant that an individual had a 1.064 chance of being classified as caries active (Table 6) The ROC analysis which was performed since the data was significant in the Univariate analysis indicated that the optimal cut-off value for the salivary Vitamin-D was 28.33 pg/ml with a sensitivity of 71% and a specificity of 57%, with AUC of 0.694.

**Table 6:** Logistic regression to establish odds ratio

	B	S.E	Wald	Df	Sig	Exp(B)	95% C.I.for EXP(B)	
							Lower	Upper
Vitamin D	-.063	.011	31.171	1	.000	.939	.919	.960
Constant	2.502	.319	61.629	1	.000	12.202		

*P* < 0.05 was considered statistically significant

Variable(s) entered on step 1: VITAMIN D

The LL-37 assay results exhibited a 7.07 ng/ µl of salivary LL-37 in the caries free group individuals, in comparison to 7.05 ng/ µl for the caries active individuals (data not statistically significant, highlighted in Table 7). It was also observed that the salivary LL-37 levels did not significantly vary with severity of caries in decay groups and was not significantly associated with the PUFA scores (Table 8).

**Table 7:** Association salivary LL-37 levels and study groups

Group		N	Mean	Std. Deviation	Median	IQR	Mann Whitney test *p* value
LL-37	Caries active	272	7.05	1.28	7.06	6.1–7.9	0.861
	Caries free	105	7.07	1.07	7.10	6.2–7.9	

*P* < 0.05 was considered statistically significant

**Table 8:** A comparison of salivary levels of LL-37 with decay groups and PUFA scores

	Parameter	Groups	Mean ± S. D	Significance (*P* Value)
Salivary LL-37(ng/ µl)	Decay Group	1	6.90 ± 1.43	0.161
		2 – 3	7.13 ± 1.18	
	PUFA Score	0	7.02 ± 1.28	0.174
		1	6.98 ± 1.31	
		2 – 5	7.65 ± 1.19	

*P* < 0.05 was considered statistically significant

The logistic regression was performed and the odds ratio depicted 1.309 effect of salivary LL-37 on caries active group. ROC analysis was performed and the optimal cut off for LL-37 is 6.81 ng/ µl with low sensitivity and specificity and area under the curve is 0.506. However, the results were not statistically significant (Table 9).

**Table 9:** Logistic regression to establish odds ratio

	B	S.E	Wald	df	Sig	Exp(B)	95% C.I.for EXP(B)	
							Lower	Upper
LL-37	.270	.286	.890	1	.345	1.309	.748	2.293
Constant	-.126	2.507	.003	1	.960	.882		

*P* < 0.05 was considered statistically significant

Variable(s) entered on step 1: LL-37

The salivary levels of IL-17A and IL-6 among the individuals in caries active and caries free groups were observed to be 155.01 ng/ml and 174.20 ng/ml respectively. However, the data was deemed to be statistically insignificant upon further analyses. It was also observed that the salivary IL-17A levels did not vary with the severity of caries in individuals nor did the PUFA scores significantly vary (Tables 10 and 11). Logistic regression was performed, and odds ratio showed salivary IL-17A 0.999 effect on caries active group. The ROC analysis that was performed exhibited an optimal cut-off of 189.9 ng/ml of IL-17a, with low sensitivity and specificity and a curve area of 0.556. The data was not statistically significant (data highlighted in Table 13). The IL-6 levels in the individuals of the caries active and caries free groups were observed to be 31.15 ng/ml and 28.33 ng/ml respectively with the data not considered to be statistically significant. It was also observed that the salivary IL-6 levels did not vary with the severity of caries or the associated PUFA scores (data highlighted in Tables 10 and 12). Logistic regression was performed, and odds ratio showed salivary IL-6 1.006 effect on caries active group. The ROC analysis that was performed exhibited an optimal cut-off of 17.60 ng/ml of IL-6 with low sensitivity and specificity, and a curve area of 0.521. However, the data was not statistically significant (highlighted in Table 13).

**Table 10:** Associating salivary IL-6 and IL-17A levels in between the study groups

Group		Mean	Std Deviation	IQR	Mann whitney test *p* value
IL-17A (ng/ml)	Caries active	155.01	91.48	99.5–198.9	0.537
	Caries free	174.20	84.77	159–221.7	
IL-6 (ng/ml)	Caries active	31.15	40.98	2.8–36.7	0.813
	Caries free	28.33	31.81	11.9–24.3	

*P* < 0.05 was considered statistically significant

**Table 11:** A comparison of salivary levels of IL-17A with decay groups and PUFA scores

	Parameter	Groups	Mean ± S. D	Significance (*P* Value)
Salivary IL-17A	Decay Group	1	143.53 ± 8.8	0.431
		2—3	161.23 ± 9.3	
	PUFA Score	0	156.24 ± 8.2	0.935
		1	156 ± 11.4	
		2—5	141.75 ± 10.2	

*P* < 0.05 was considered statistically significant

**Table 12:** A comparison of salivary levels of IL-6 with decay groups and PUFA scores

	Parameter	Groups	Mean ± S. D	Significance (*P* Value)
Salivary IL-6	Decay Group	1	29.44 ± 36.59	0.794
		2 – 3	32.07 ± 43.52	
	PUFA Score	0	30.68 ± 38.89	0.873
		1	29.65 ± 40.65	
		2 – 5	39.49 ± 63.11	

*P* < 0.05 was considered statistically significant

**Table 13:** Logistic regression to establish odds ratio

	B	S.E	Wald	df	Sig	Exp(B)	95% C.I. for EXP(B)	
							Lower	Upper
IL-17A	-.001	.004	.115	1	.735	.999	.991	1.007
IL-6	.006	.010	.366	1	.545	1.006	.987	1.026
LL-37	.270	.286	.890	1	.345	1.309	.748	2.293
Constant	-.126	2.507	.003	1	.960	.882		

*P* < 0.05 was considered statistically significant

## Discussion

Past research has shown that genetic factors play a vital role in risk of dental caries, which in turn is due to the multifaceted nature of caries itself [16]. Vitamin D has been shown to control calcium haemostasis which in turn significantly influences immune responses and anti-inflammatory activity [17]. Studies have also noted that bone phenotype, hormonal balance, food, and sun exposure may all play a vital role in the variation of vitamin D receptor gene polymorphism that is observed among different races and age groups [18–20]. In the current study, the practices of individuals in both caries free and caries active groups were similar e.g. brushing teeth once a day, no significant food intake in between meals, and low intake of sugary or sticky food items. Therefore, these factors were not considered for the study. Research has shown that environmental and underlying genetic factors are associated with various other factors that cause development of dental caries [21]. It has also been shown that susceptibility to caries may differ from individual to individual, even though an individual may be considered as high risk of developing caries [22].

In the current study, salivary vitamin D levels were observed to be significantly higher in the caries-free group as compared to the caries active group. This can be attributed to the production of protective peptides (LL-37/cathelicidins) following their activation via the TLR2-vitamin D LL-37 mechanism, where the production of these peptides occur as a result of the binding of 1,25(OH)2 D to the Vitamin D receptor. The LL-37 has been noted to possess the potential for increasing the antimicrobial capacity of anti-inflammatory cells like neutrophils [23]. In the current study, the elevated levels of salivary vitamin D in the caries free group exhibit the effectiveness of vitamin D to play an antibacterial role by regulating the production of these naturally occurring peptides. Vitamin D has also been noted to upregulate numerous proteins such enamelin, dentin sialoproteins, amelogenins, and dentin phosphoproteins, while also stabilizing the demineralization and disintegration of tooth surface while preserving the appropriate surface proteins [24]. In the current study, the salivary vitamin D levels among participants in both the groups could be linked to normal to average sun exposure and to a variety of dietary sources. A past study by Gyll et al. evaluated the association of dental caries and salivary vitamin D levels post vitamin D supplementation, and noted high vitamin D levels in individuals without caries [25]. A similar study conducted by Chhonkar et al. exhibited that vitamin D was an important factor in preventing dental caries. Studies have also shown that absence of caries can be attributed to the role of vitamin D in the production of LL-37 peptides via the TLR2-Vitamin D pathway [26, 27], which is in accordance with the results of the present study where we evaluated the protective role of vitamin D levels in dental caries progression and prevalence.

Antimicrobial peptide LL-37 was evaluated in the current study for both caries active and caries free individuals, with the results exhibiting that the levels of LL-37 were higher in individuals without caries as compared to those having caries (not statistically significant). LL-37 has been shown to reduce biofilm formation on the tooth surface, reduce thickness of existing biofilms, and decreasing the adherence of microbes onto the tooth surface, thereby decreasing the production of inflammatory markers [28]. Similarly, another study evaluated the LL-37 levels in children wherein it was noted that lower levels were associated with higher caries activity, albeit statistically insignificant. The same study also noted that LL-37 had the potential to be a prognostic marker against caries in children, adolescents, and adults [29, 30]. LL-37 has been observed in the past in the carpet, toroidal, and barrel stave models to have potency against Streptococcus mutans by preventing growth and colonization [28]. Another past study showed that the direct effect of the LL-37 peptide was to cause enzyme mediated destruction of bacteria while the indirect effect was to regulate inflammatory markers [29]. In another study, the production and biochemical levels of cathelicidins were noted to be directly affected by the levels of inflammation and vitamin D [31].

The current study also evaluated the IL-6 levels among the individuals as noted an increase in the caries active group in comparison to the caries free group (data statistically insignificant). This could be as a result of the pro-inflammatory function of the IL-6 interleukin. Studies have shown that IL-6 is a key factor in the inflammatory response since it activates neutrophil proliferation at the inflammatory sites. The study also noted that IL-6 plays a vital role in the pathology of diseases due to its pleitropy, role in immunosenescence, and caries formation [32] therefore the IL-6 may be a potential indicator for inflammation in oral cavity, however needs to be validated with more supporting studies. Apart from IL-6, IL-17A levels were also analyzed in the individuals participating in the current study and it was observed that IL-17A levels were higher in those individuals without caries (data statistically insignificant). This could have been due to the levels of LL-37 maintaining inflammatory balance to promote repair in individuals with caries. Past studies have shown that immune processes, both innate and adaptive, affect dental biofilm formation, which in turn affect caries formation. However, the studies were limited to evaluating a single nucleotide polymorphism in the vitamin D receptor and the polymorphisms in the CAMP gene, another factor affecting LL-37 levels in saliva, were excluded from the scope [33–35].

## Conclusions

The present study focused on evaluating the levels of Vitamin D, IL-17A, IL-6, and LL-37 in saliva of individuals with and without caries. Salivary vitamin D was higher in caries free individuals as compared to those with caries. This could be because vitamin D plays an important role in preventing caries by activating enzymes which in turn convert 25, hydroxyl vitamin D to 1,25-dihydroxy vitamin D. This in turn binds to vitamin D binding protein to form a complex to activate the LL-37 peptides via binding to vitamin D receptors. The current study noted that LL-37 was higher in caries free individuals but not statistically significant in comparison to individuals with caries, possibly due to LL-37’s role in preventing and neutralizing biofilms and bacterial colonization to hinder caries formation. Interleukins IL-6 and IL-17A were also higher in caries free individuals but not statistically significant from those with caries. This could be attributed to the pro-inflammatory activity of IL-6 and the regulating role of LL-37 in IL-17A production to promote repair respectively. Therefore it can be said that all three biochemical markers could be used as a prognostic marker to predict incidence of caries in individuals.

## Data Availability

The datasets used and/or analysed during the current study available from the corresponding author on reasonable request.
